# Moxifloxacin Increases Heart Rate in Humans

**DOI:** 10.3390/antibiotics6010005

**Published:** 2017-02-05

**Authors:** Jay W. Mason, Thomas E. Moon

**Affiliations:** 1 Department of Medicine, University of Utah School of Medicine, Salt Lake City, UT 84132, USA; 2 Tarizona eHealth Services, Tucson, AZ 85732, USA; tmoonnew@gmail.com

**Keywords:** moxifloxacin, heart rate, thorough QT study

## Abstract

(1) *Background*: We assessed the effect of moxifloxacin on heart rate, and reviewed the heart rate effects of other antibiotics; (2) *Methods*: A total of 335 normal volunteers had 12-lead electrocardiograms recorded at multiple time points before and during treatment with moxifloxacin and with placebo in seven consecutive, thorough QT studies of crossover design; (3) *Results*: The average baseline heart rate across the seven studies was 61.5 bpm. The heart rate after moxifloxacin dosing was analyzed at five time points shared by all seven studies (hours 1, 2, 3, 12 and 24). The maximum mean heart rate (HR) increase for the seven studies combined was 2.4 bpm (95% CI 1.6, 3.3) at hour 2. The range of mean maximum increases among the seven studies was 2.1 to 4.3 bpm. For the seven studies combined, the increase was statistically significant at all but the 24 h time point. The maximum observed individual increase in HR was 36 bpm and the mean maximum increase was 30 ± 4.1 bpm by time point and 8 ± 6.9 bpm by subject. Many antibiotics increase HR, some several-fold more than moxifloxacin. However, clinicians and clinical investigators give little attention to this potential adverse effect in the medical literature; (4) *Conclusions*: The observed moxifloxacin-induced increase in HR is large enough to be clinically relevant, and it is a potentially important confounder in thorough QT studies using moxifloxacin as an active control. More attention to heart rate effects of antibiotics is warranted.

## 1. Introduction

Antibiotics are generally not thought to affect heart rate (HR) in humans. However, there is modest evidence in animal models that macrolide antibiotics [1,2,3,4,5,6] and, to a lesser extent, fluoroquinolone antibiotics [7] may increase HR, though there is only sparse clinical evidence for an HR effect in humans for these antibiotic classes [8,9]. The fluoroquinolone moxifloxacin is of particular interest because it is often used to treat infections associated with an already-increased HR, such as community-acquired pneumonia [10], and it is used as an active control in thorough QT (TQT) studies [11,12] QT is the electrocardiographic QT interval and reflects the duration of myocardial repolarization. TQT studies are rigidly designed protocols to measure QT interval changes caused by drugs. 

The purpose of this report is to determine if moxifloxacin affects HR in normal subjects, to consider its implications for clinical care and clinical research and to summarize the HR effects of other antibiotics known to increase HR. This first objective was achieved by determining the HR change induced by moxifloxacin in seven thorough TQT studies. TQT studies are currently required for approval of most new drugs to assess the risk of QT prolongation, in accordance with the ICH E-14 Guidance [13]. We also examined changes in the PR interval.

## 2. Results

The details of each study are summarized in Table 1. Each study included from 41 to 50 subjects, 335 in all. The mean age ranged from 30 to 36 years. More men than women were enrolled (191 vs. 144). The average HR among the five studies was 61.5 beats per minute (bpm) at the pre-drug morning baseline.

Table 2 shows the baseline- and placebo-subtracted unadjusted and mixed model–adjusted mean changes in HR (ddHR) with their 95% two-tailed confidence intervals (CI) at each time point for each study and all studies combined after the administration of moxifloxacin. The similarity of means in the unadjusted data and the mixed model and the narrower confidence bands in the mixed model are consistent with a successful model.

**Figure 1:**
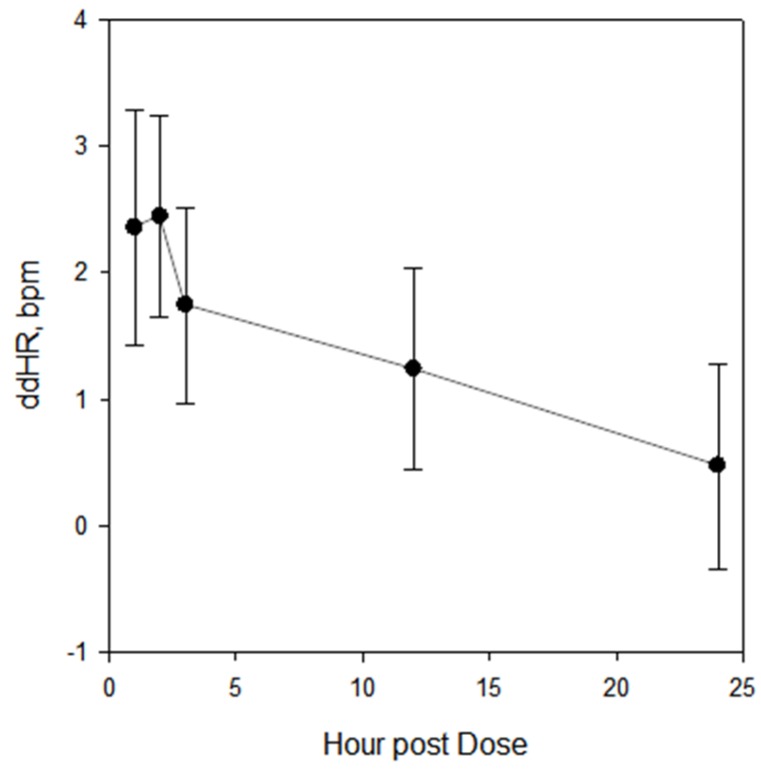
Mean ddHR and 95% CI, all seven studies combined. An HR increase was present and maximal at 2 h after administration of moxifloxacin. HR then decreased during the ensuing 22 h. The lower 95% confidence interval (CI) exceeded 0 at all but the last time point.

In the analysis of all studies combined, the change in HR was positive at all time points and the change was statistically significant, as judged by exclusion of zero by the confidence boundaries, at all time points except for the last time point (24 h) in both the unadjusted and mixed models. In the individual studies, the mean HR change was positive at most time points (32 of 35 in the unadjusted model and 33 of 35 in the mixed model) and the change was statistically significant at one or more time points in each of the studies in both models. The maximum mean change of HR in the combined analysis occurred at hour 2, as shown in Figure 1. ddHR then decreased through hour 24.

The maximum increase in ddHR observed in individuals at any time point was 36 bpm and the mean maximum increase at any time point for all subjects was 30 ± 4.1 bpm. The mean maximum averaged across all time points by subject was 8 ± 6.9 bpm. Forty-eight subjects (14%) experienced a maximum increase of at least 15 bpm, and 107 (32%) experienced an increase of 10 bpm or more.

The mixed effect model showed a significant relationship between the ddHR and hour (*p* < 0.0001), but study (*p* = 0.4034), sex (*p* = 0.3192) and age (*p* = 0.3062) were not influential.

**Figure 2:**
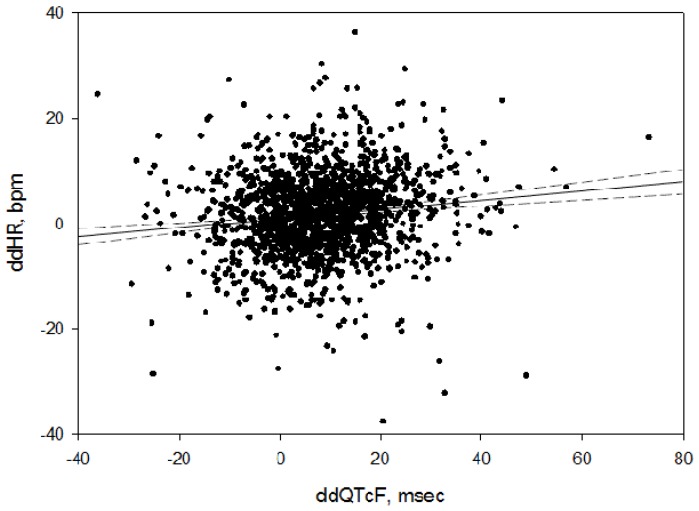
Bivariate fit of ddHR to ddQTcF. The straight black line is the fit line, with a ddHR intercept of 0.9 and a slope of 0.08. The dashed lines represent the 95% confidence interval of the fit.

Since there is a known concentration-response relationship for moxifloxacin and ddQTcF [14] (baseline and placebo subtracted QT, corrected for HR by the Fridericia formula [15]), the relationship between ddHR and ddQTcF was examined in a simple linear regression, with ddQTcF serving as a surrogate for the moxifloxacin concentration, which was not available in this analysis. As shown in Figure 2, there was a significant positive relationship (ddHR = 0.93 + 0.08 × ddQTcF, *p* < 0.0001, *R^2^* = 0.017). The formula indicates that ddHR, bpm, can be predicted by adding 0.93 to the product of ddQTcF times 0.08.

The electrocardiographic PR interval was also significantly affected by moxifloxacin, decreasing by 3.5 msec (95% CI −4.7, −2.3).

## 3. Discussion

Our analysis demonstrates that moxifloxacin caused an increase in HR after a single 400 mg dose in normal subjects. The average peak ddHR among the 335 subjects of this study was substantial: 8 bpm, with 32% experiencing an increase greater than 10 bpm. Age and sex did not influence the change in HR. This effect of moxifloxacin was not previously recognized.

Though several antibiotics of two distinct classes are now known to increase HR in humans (see Table 3 and discussion below), it appears that this effect is usually overlooked in the choice of antibiotics for the treatment of infection.

None of the package inserts for the approved drugs listed in Table 3 include information or precautionary language regarding dose-related cardiac acceleration. Our literature search found no citations that include “antibiotic” and “heart rate” or “tachycardia” in the title. Substituting macrolide or fluoroquinolone for antibiotic in the search also yielded no citations. Searching for these same strings in the abstract did yield citations, but none of them dealt with the specific phenomenon of post-dosing HR increase in humans. There is essentially no information on the extent to which this change occurs in patients treated for infections and it is not known if this potentially adverse effect has had significant clinical consequences. In recent years, two investigative groups have found an association between macrolide antibiotics and sudden death [21,22]. However, in both cases the investigators ascribe the relationship to either direct QT prolongation or QT prolongation related to metabolic inhibition. Neither group considered HR effects as potentially causative, and neither mentioned the phenomenon.

Four of the seven drugs listed in Table 3 (erythromycin, levofloxacin, telithromycin and moxifloxacin) have been in clinical use for many years. Their HR effects in normal volunteers are relatively small. That and the improvement in baseline tachycardia following antibiotic administration probably account for the phenomenon being unrecognized clinically. It is also possible that patients with fever-induced cardiac acceleration are not vulnerable to antibiotic-related HR increase. A study by Haverkamp and colleagues [23] is consistent with this possibility, in that a summary of 64 trials of moxifloxacin in patients treated for infection revealed a modest decrease in heart rate (about 2 bpm) at presumed Cmax in moxifloxacin recipients. It is also possible that the salutary effects of the antibiotic were already present and blunted the cardiac acceleratory effect of moxifloxacin.

The mechanisms responsible for the HR increase induced by moxifloxacin, as well as by the other antibiotics in Table 3, are unknown. An autonomic effect (sympathetic stimulation, vagal withdrawal, or both) is a likely possibility. In a study performed in guinea pigs anesthetized with pentobarbital, Wisialowski and colleagues [24] found a modest decrease in heart rate associated with moxifloxacin, erythromycin and telithromycin infusion. Pentobarbital is well known to strongly influence the autonomic nervous system, reducing heart rate, blood pressure and baroreflex responses [25]. The absence of a heart rate increase in this study supports an autonomic mechanism in unanesthetized humans. An alternative to an autonomic mechanism for antibiotic-related cardiac acceleration might be the alteration of an ionic current shared by the sinus and atrioventricular nodes. One such possibility is stimulation of the pacemaker current, I_f_ [26]. Though I_f_ density is lower in the atrioventricular node than in the sinus node, the I_f_ blocker ivabradine does prolong the PR interval at clinical doses [27]. Thus, an agonist effect on I_f_ might be expected to change both HR and PR. Likewise, an agonist effect on L-type calcium channels could have produced the combined HR and PR changes seen in this study. Moxifloxacin is not known to affect either I_f_ or I_Ca,L_, though josamycin and erythromycin have been shown to inhibit transmembrane calcium flux [6].

Main et al. [4] studied the effects of two doses of tilmicosin, a veterinary macrolide, in anesthetized dogs on inotropy, blood pressure and HR, and showed a large dose-related reduction in inotropy (left ventricular dP/dt) and mean aortic blood pressure and a large dose-related increase in HR (80 bpm), using high doses considered potentially toxic. The increase in HR was not blocked by dobutamine, despite improvement in inotropy and blood pressure, and propranolol did not blunt the tachycardia. These latter findings suggest that sympathetic mediation may not be involved.

Moxifloxacin plasma concentration data were not available for this analysis. Though moxifloxacin pharmacokinetic sampling is routinely performed in TQT studies, the samples are usually not analyzed unless the moxifloxacin effect is atypical. However, the observed time course of the effect of moxifloxacin on HR is consistent with a concentration-related effect based on the known pharmacokinetics of the drug [14]. Furthermore, using the change in ddQTcF as a surrogate for the plasma concentration of moxifloxacin, based upon their strong concentration-response relationship [14], we observed a significant positive relationship between ddHR and ddQTcF, supporting the likelihood that that the changes in HR were concentration-dependent.

The HR effect of moxifloxacin has both clinical and regulatory significance. Clinicians should be aware of its potential to increase HR substantially in normal subjects, as this same effect in patients with infection-related sinus tachycardia and comorbidities limiting their ability to adjust to an increase in HR, such as patients with heart failure, coronary ischemia, critical aortic valve stenosis and compromised cerebral or peripheral arterial circulation, could be detrimental, especially if infection control and defervescence are not achieved. Further investigation to determine if an HR increase occurs in the clinical care setting secondary to moxifloxacin or the other drugs listed in Table 3 is needed. The possibility that additional antibiotics not listed in the table share this property could be explored by clinical investigators aware of this possibility.

Though the average HR effect of moxifloxacin is modest, it may affect the accuracy of detection of moxifloxacin′s effect on QTc (QT corrected for HR) in TQT studies. In these studies, moxifloxacin was used to determine if the study design and methodologies were capable of detecting its known effect on QTc. However, in the presence of a moxifloxacin-related increase in HR, assay sensitivity is more difficult to prove because the Fridericia HR correction [15], the most commonly applied correction in TQT studies, under-corrects for an increase in HR above 60 bpm [28] The mean baseline HR in most of the seven TQT studies in this report was above 60 bpm, and it was above 60 bpm in all seven studies during moxifloxacin treatment. Since QTc is calculated on a per-subject basis, from which group mean QTc values are calculated at each time point, large HR increases in individuals, similar to what we observed in this analysis, could substantially distort the group mean QTc values, resulting in failure to meet assay sensitivity criteria. Investigators should be aware of this and consider using corrections that do not underestimate QT duration after an increase in HR.

In summary, a 400 mg oral dose of moxifloxacin increases HR in normal subjects. The increase could be large enough to influence clinical stability in some patients receiving moxifloxacin for bacterial infections, and it could lead to inaccurate assessment of assay sensitivity in TQT studies. These potential problems can be mitigated by awareness of clinicians and investigators of the effects on HR of moxifloxacin and other antibiotics.

## 4. Materials and Methods

ECG (electrocardiogram) data from seven TQT studies were combined for this analysis. Each study was designed in accordance with the ICH-E-14 regulatory guidance [13,29] and were very similar in design. Each study was performed at Spaulding Clinical Research (West Bend, WI, USA), and each study was approved by the Chesapeake Institutional Review Board (IRB). All study participants signed an informed consent document approved by the IRB. All seven studies of crossover design completed in the past four years at Spaulding Clinical Research for which treatment allocation was available and ECGs were available at hours 1, 2, 3, 12 and 24 after dosing were included in the analysis. The selection was restricted to crossover designed studies to allow for reliable subtraction of the placebo effect. For inclusion subjects were adults under the age of 60 without known clinical disorders. Subjects with bradycardia, tachycardia and QTcF prolongation, and those with relatives with QTcF prolongation, were excluded. The crossover was accomplished using a Williams′ latin square design in each study. Investigators and subjects were blinded to treatment. As shown in Table 1, each study had three or four treatment arms: placebo, moxifloxacin and one or two doses of the investigational drug. Only the placebo and moxifloxacin data were analyzed for this report.

Each electrocardiogram was recorded by a Mortara Surveyor 12-lead telemetry system (Mortara Instrument, Milwaukee, WI, USA). After automated interval measurement and diagnostic statements were affixed to each recording by the Mortara Veritas algorithm, it was submitted to a cardiologist for over-reading in an electronic workstation (Mortara Instrument, Milwaukee, WI, USA). Each individual′s ECGs were assigned to a single cardiologist who had no knowledge of treatment assignment or sequence. The readers reviewed and adjusted all ECG intervals analyzed in this study (RR (a reciprocal of HR), PR and QT). QT was corrected for its dependence on HR using the Fridericia formula [15].

Baseline recordings were obtained in triplicate at three separate morning time points from 90 min before drug administration to time 0 in two of the studies (studies 1 and 5) and at a single time point immediately before time 0 in the other five studies. A single set of triplicate ECGs was recorded at all subsequent time points.

Moxifloxacin was administered as a single oral dose of 400 mg at time zero. Moxifloxacin and placebo were over-encapsulated for the purpose of blinding, using a standardized technique which was not found to influence pharmacokinetics of moxifloxacin [14]. In all seven studies subjects were fasted prior to and for at least 2 h after drug administration.

Analyses were restricted to time points represented in all seven studies (pre-dose and hours 1, 2, 3, 12 and 24) to avoid potential bias introduced by time points unique to individual studies. In the moxifloxacin arms 1551 ECGs were recorded prior to dosing and were averaged to yield the 335 sets of pre-dose time point values used in the analysis. For the five post-dose time points, a total of 5025 ECGs yielded 1675 sets of time point values. As the same number of ECGs were used to generate placebo results, a total of 13,152 ECGs contributed to the results of this analysis. The primary analysis was carried out on ddHR (double delta HR, calculated by subtracting the pre-dose baseline HR from heart rates for each subject at each on-treatment time point and then subtracting the placebo baseline-adjusted HR from the moxifloxacin baseline-adjusted HR. A similar analysis was performed on the baseline and placebo-subtracted change in PR (ddPR).

Descriptive and inferential statistical analysis was performed with JMP version 9.03 (SAS Institute, Cary, NC, USA). The linear mixed effects model included ddHR as the dependent variable, subject as a random effect and study, hour, age and sex as fixed effects.

Literature reviews were conducted using EndNote on PubMed. The FDA website was also searched for antibiotics with heart rate effects.

## 5. Conclusions

Moxifloxacin at standard clinical dosage induces a modest increase in HR which can be substantial in some individuals. From a clinical perspective, this effect should be taken into account by physicians prescribing antibiotics in patients that might be vulnerable to tachycardia. It should also be taken into account by clinical investigators using moxifloxacin as an active control in TQT and similar research studies. There are six additional antibiotics known to increase HR, as listed in Table 3. The same precautions apply to these and any other antibiotics that may be found in the future to accelerate the heart.

## Figures and Tables

**Table 1:** Description of the seven clinical studies.

Study	N Subjects	Mean Age (Years)	Sex (% M)	Mean Baseline Pre-Dose HR	Treatments
1	50	31 ± 8.7	60	61.8 ± 9.2	Placebo, moxifloxacin, study drug dose 1, study drug dose 2
2	49	31 ± 9.0	57	57.7 ± 9.1	Placebo, moxifloxacin, study drug dose 1, study drug dose 2
3	53	30 ± 8.0	42	63.6 ± 9.8	Placebo, moxifloxacin, study drug
4	48	36 ± 9.1	50	59.8 ± 8.6	Placebo, moxifloxacin, study drug dose 1, study drug dose 2
6	47	36 ± 9.7	67	60.4 ± 9.4	Placebo, moxifloxacin, study drug
5	41	32 ± 8.2	63	64.5 ± 9.6	Placebo, moxifloxacin, study drug
7	47	32 ± 10.0	64	60.0 ± 8.4	Placebo, moxifloxacin, study drug dose 1, study drug dose 2

N subjects = number of subjects receiving moxifloxacin; M = male; HR = heart rate in bpm.

**Table 2:** ddHR by study and time point.

Study	Time Point, Hour	Unadjusted (bpm)		Mixed Model (bpm)	
		ddHR	95% CI	ddHR	95% CI
All	1	2.4	1.5, 3.2	2.4	1.6, 3.2
	2	2.4	1.6, 3.3	2.5	1.7, 3.3
	3	1.7	0.9, 2.6	1.8	1.0, 2.6
	12	1.2	0.4, 2.1	1.3	0.4, 2.1
	24	0.5	−0.4, 1.3	0.5	−0.3, 1.3
1	1	2.1	0.3, 4.0	2.2	0.4, 4.1
	2	0.8	−1.1, 2.6	0.9	−1.0, 2.8
	3	1.0	−0.8, 2.9	1.1	−0.8, 3.0
	12	−0.0	−1.9, 1.9	0.1	−1.8, 2.0
	24	−0.7	−2.5, 1.2	−0.6	−2.5, 1.3
2	1	2.5	0.1, 4.9	2.4	−0.0, 4.8
	2	2.4	−0.0, 4.8	2.3	−0.1, 4.7
	3	1.7	−0.7, 4.1	1.6	−0.8, 4.0
	12	1.5	−0.9, 3.9	1.4	−1.0, 3.8
	24	0.5	−1.9, 2.9	0.4	−2.0, 2.8
3	1	1.0	−1.5, 3.5	1.0	−1.6, 3.5
	2	3.2	0.7, 5.7	3.2	0.6, 5.7
	3	2.5	0.1, 5.0	2.5	−0.0, 5.1
	12	0.6	−1.9, 3.1	0.6	−2.0, 3.1
	24	1.3	−1.2, 3.8	1.3	−1.3, 3.8
4	1	4.3	2.6, 6.0	4.3	2.7, 6.0
	2	3.6	1.9, 5.3	3.6	2.0, 5.3
	3	2.9	1.2, 4.6	2.9	1.2, 4.5
	12	3.5	1.8, 5.2	3.5	1.8, 5.1
	24	1.7	0.0, 3.4	1.7	0.1, 3.4
5	1	2.1	−0.1, 4.3	2.0	−0.3, 4.3
	2	3.0	0.8, 5.2	2.9	0.6, 5.3
	3	1.4	−0.8, 3.6	1.3	−1.0, 3.6
	12	1.1	−1.1, 3.3	1.0	−1.3, 3.3
	24	−1.0	−3.2, 1.2	−1.3	−3.7, 1.0
6	1	2.4	0.3, 4.5	2.7	0.5, 4.9
	2	1.5	−0.7, 3.6	1.8	−0.4, 3.9
	3	0.3	−1.8, 2.4	0.6	−1.5, 2.8
	12	0.7	−1.4, 2.9	1.0	−1.1, 3.2
	24	0.0	−2.1, 2.1	0.3	−1.9, 2.5
7	1	2.3	−0.1, 4.6	2.5	0.0, 5.0
	2	2.5	0.2, 4.8	2.8	0.3, 5.2
	3	2.1	−0.2, 4.5	2.4	−0.1, 4.9
	12	1.3	−1.0, 3.6	1.6	−0.9, 4.0
	24	1.3	−1.0, 3.6	1.6	−0.9, 4.1

**Table 3:** Antibiotics that increase heart rate.

Drug	Mean Maximum HR Increase	Dose	Antibiotic Class	Comment
Cethromycin [16]	4.4 bpm 11.3 bpm	300 mg po 900 mg po	Macrolide (ketolide)	Not approved
Erythromycin [9]	4 bpm	500 mg iv	Macrolide	Approved
Levofloxacin [17]	8 bpm	1500 mg po	Fluoroquinolone	Approved
Levonadifloxacin [18]	15 bpm	2600 mg po	Fluoroquinolone	In development
Moxifloxacin (this study)	2.4 bpm	400 mg po	Fluoroquinolone	Approved
Solithromycin [19]	15 bpm	800 mg iv	Macrolide (ketolide)	In development
Telithromycin [20]	13 bpm	3200 mg	Macrolide (ketolide)	Approved

## Supplementary Materials

Supplementary File 1
